# Supplementary material for: From qualitative data to correlation using deep generative networks: Demonstrating the relation of nuclear position with the arrangement of actin filaments
Source: PLoS One. 2022 Jul 29;17(7):e0271056. doi: 10.1371/journal.pone.0271056 (PMC9337686; doi:10.1371/journal.pone.0271056)
Supplement: S1 File — The cell image database used in this research is available in the supplementary file “NIH3T3_ImageDataset-20220519T022044Z-001.zip”. The network source code can be found at: https://github.com/JGFermart/NuclearPrediction. (PDF) [file pone.0271056.s001.pdf]

Supplementary Information

From qualitative data to correlation using deep generative networks: Demonstrating the relation of nuclear position with the arrangement of actin filaments

Jyothsna Vasudevan, Chuanxia Zheng, James G. Wan, Tat-Jen Cham, Lim Chwee Teck & Javier G. Fernandez*

* To whom correspondence should be addressed. E-mail: javier.fernandez@sutd.edu.sg

This material includes:

Figure 1 TFill Network performance assessment
Figure 2 Generation of nuclei and matching
Figure 3 Nucleus distribution in the image and cell

Figures

a

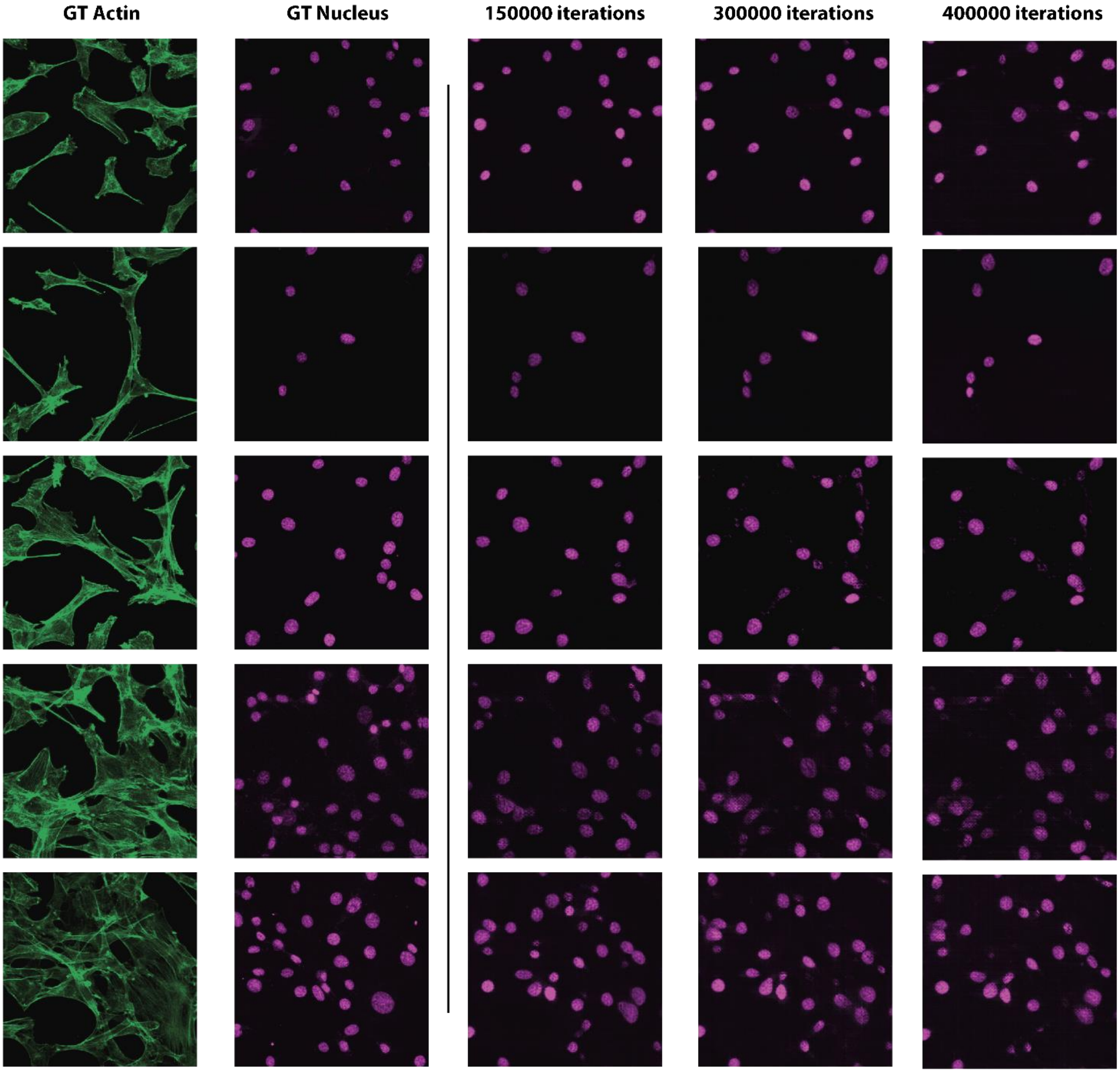

b

Model	Iterations	l1loss ↓	SSIM ↑	PSNR ↑	LPIPS ↓	FID ↓
TFill	150000	0.0117 ± 0.0002	0.8333 ± 0.0175	32.9127 ± 65.3693	0.1204 ± 0.0105	32.77
	300000	0.0117 ± 0.0002	0.8328 ± 0.0174	33.1595 ± 65.2648	0.1209 ± 0.0092	21.66
	**400000**	**0.0114 ± 0.0002**	**0.8335 ± 0.0165**	**33.5130 ± 68.2507**	**0.1171 ± 0.0089**	**14.28**

Fig. 1| Iterative Evaluation of TFill Neural Network Performance. a, Visual comparison of TFill generated nuclei images with altering training iterations; **b,** Quantitative comparison of TFill generated images with altering training iterations using various metrics from computer vision (↓ Lower is better; ↑ Higher is better)

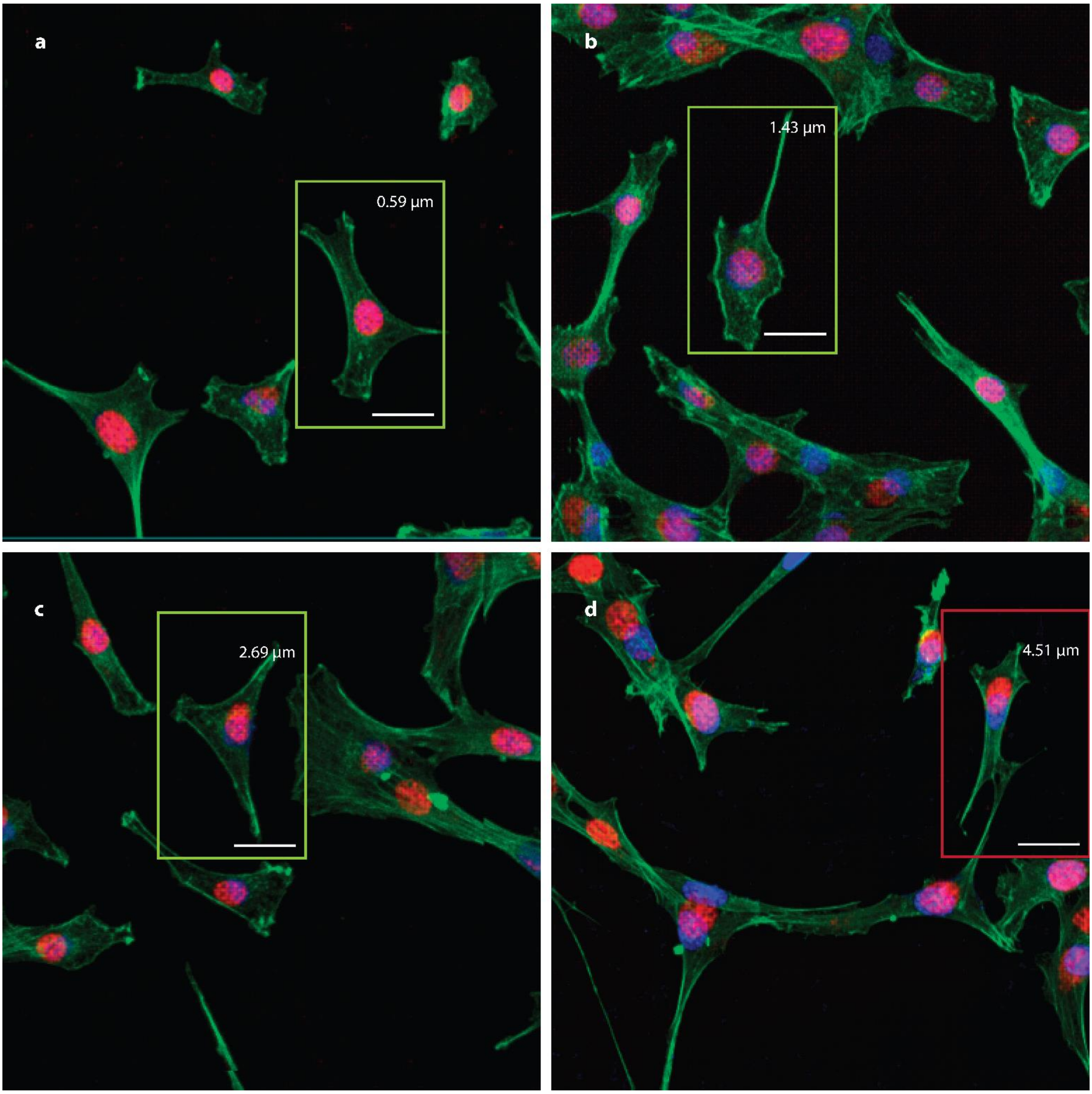

Fig. 2| Generation of nuclei and matching. Full images of panels in Figure 3c of the main text. The generated nuclei (red) and real nuclei (blue) have been placed over the images of the actin fibers used for their generation. Bars are 20 µm, and the values correspond to the distance between the centroids of the generated and real nuclei (i.e., ground truth) of the framed cell.

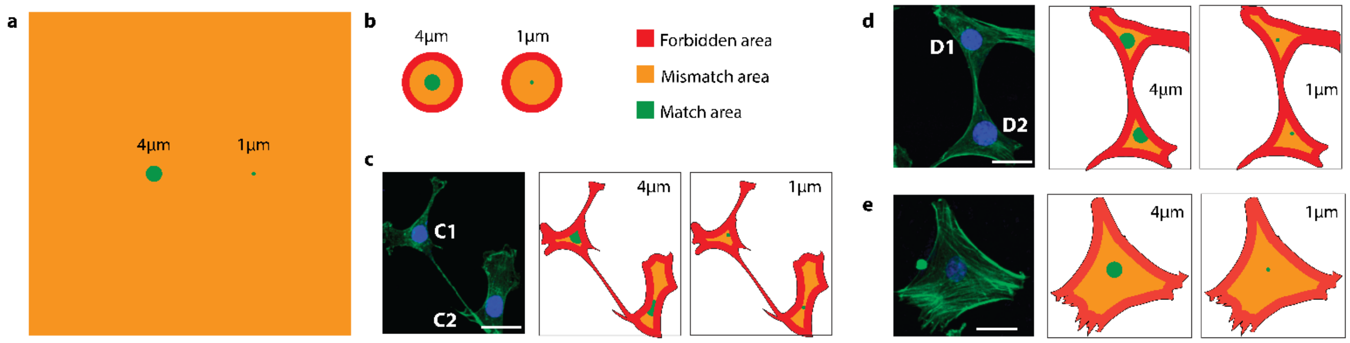

	4 µm (5785/8151)				1 µm (2328/8151)			
	Match	Mismatch	Z-score	p-value	Match	Mismatch	Z-score	p-value
A- Full image (159.41x159.41µm)	0.001978	0.998022	1438.124	0	0.000124	0.999876	2318.232	0
B- Modeled circular cell (Nucelus diameter 8µm, cell diameter 30µm)	0.132231	0.867769	153.9169	0	0.008264	0.991736	276.5795	0
C1 – Extreme narrow (top)	0.580192	0.419808	23.69674	0	0.071678	0.928322	74.87525	0
C2- Regular cell (bottom)	0.110951	0.889049	172.1246	0	0.019449	0.980551	174.0065	0
D1- Regular cell (top)	0.29502	0.70498	82.09835	0	0.021562	0.978438	164.1266	0
D2- Regular cell (bottom)	0.301934	0.698066	80.19432	0	0.024732	0.975268	151.6532	0
E- Large cell	0.059372	0.940628	248.4612	0	0.003778	0.996222	414.7504	0

Fig. 3| Nucleus distribution in the image and cell. In all panels, green refers to those areas where positions the nuclei centroid is considered right (Match). Orange areas are possible positions for the centroid considered a failure (Mismatch), and red areas are those positions forbidden for the nucleus centroid for being too close to the cell edge. **a,** Distribution of possible positions for the whole microscope image. The green area are those positions at either 4 or 1µm away from the centroid of the real nucleus. **b,** Represent the distribution in a circular model of a cell using the average radius of a cell in confluence. **c-e,** Examples of the distribution of positions in cells used in this study. Cells C1 and D represent the highest and lowest ratios found. The bottom table is a calculation of the p-value for all the possible distributions. A p-value<0.05 is commonly considered enough to discard the null hypothesis and support the alternative hypothesis. In this case, the null hypothesis is a random distribution of the nuclei with respect to the actin fibers. The alternative hypothesis is the deterministic relation of the nucleus position with the fiber arrangement. In all cases, p ranges between 10^{-100} and 10^{-2200} and have been approximated to 0.
